# Supplementary figures and images for: GWAS Reveals Stable Genetic Loci and Candidate Genes for Grain Protein Content in Wheat
Source: Curr Issues Mol Biol. 2025 Nov 25;47(12):981. doi: 10.3390/cimb47120981 (PMC12731824; doi:10.3390/cimb47120981)

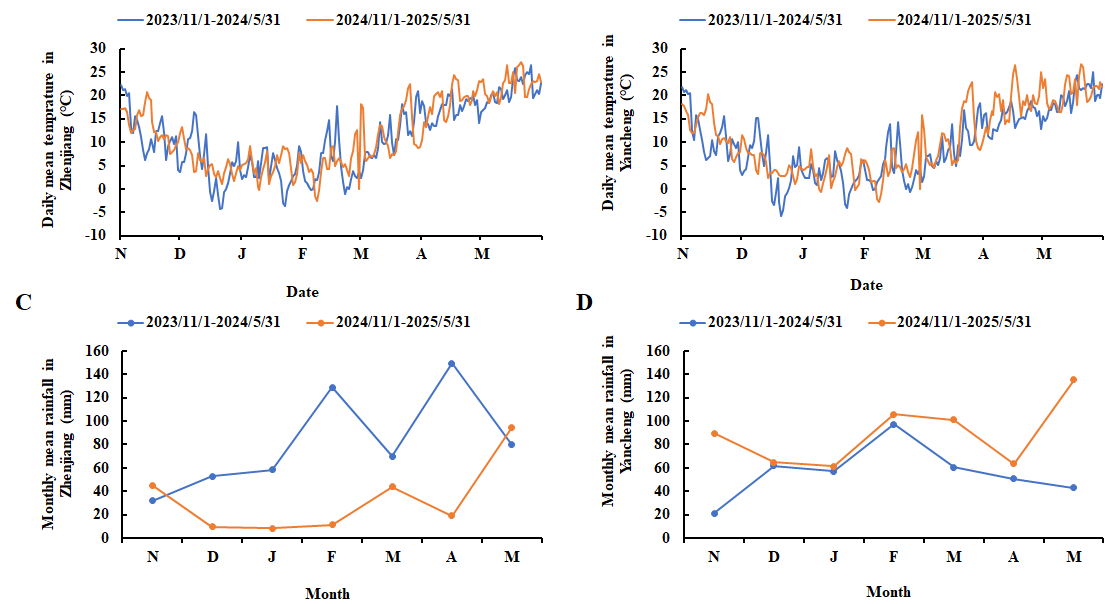

Supplement: Supplementary file 1 [file cimb-47-00981-s001.zip › Figure S1.tif]

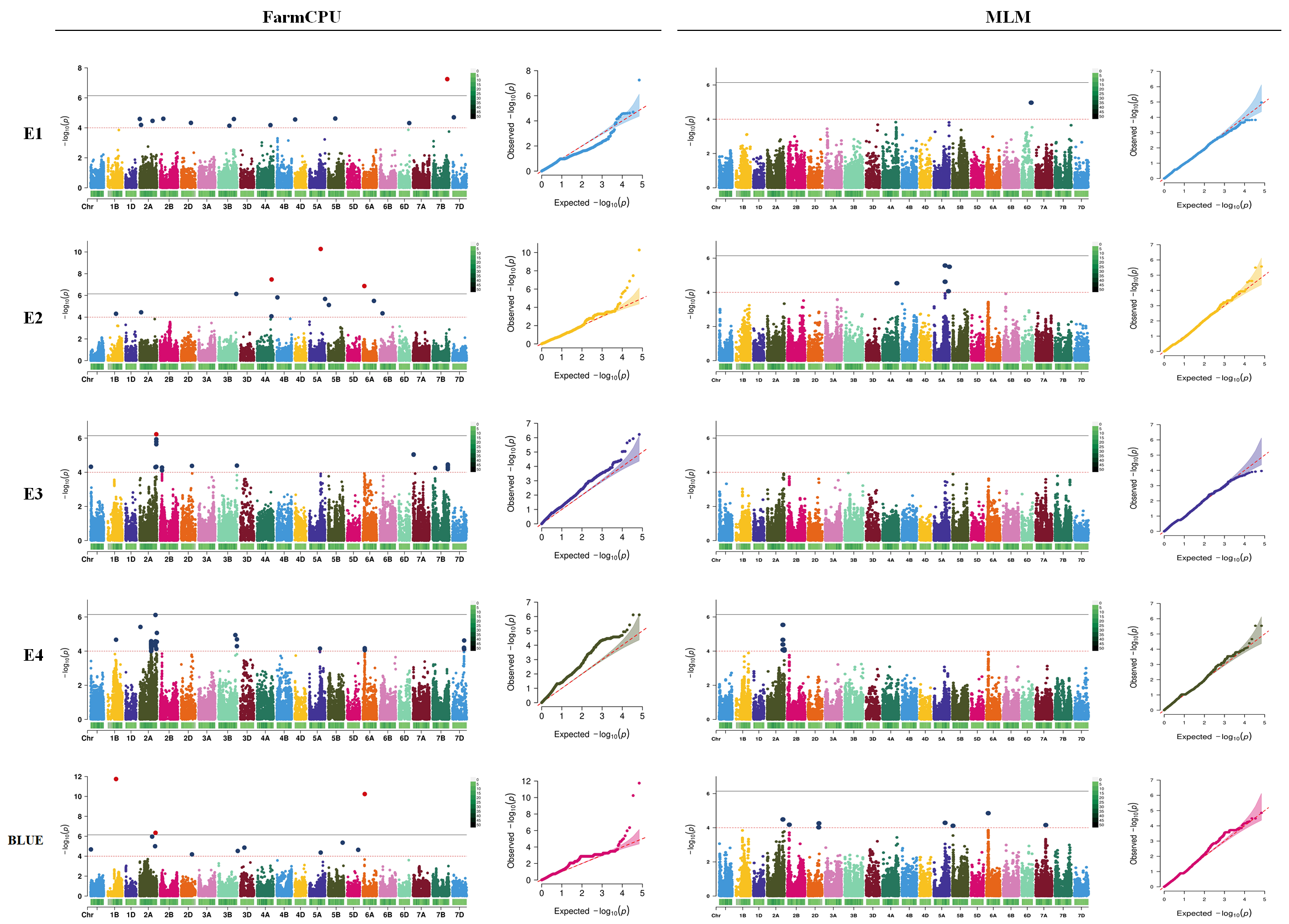

Supplement: Supplementary file 1 [file cimb-47-00981-s001.zip › Figure S2.tif]
